# RNAi silencing of wheat gliadins alters the network of transcription factors that regulate the synthesis of seed storage proteins toward maintaining grain protein levels

**DOI:** 10.3389/fpls.2022.935851

**Published:** 2022-08-08

**Authors:** Miriam Marín-Sanz, Francisco Barro

**Affiliations:** Department of Plant Breeding, Institute of Sustainable Agriculture (IAS), Spanish National Research Council (CSIC), Córdoba, Spain

**Keywords:** RNA-seq, prolamin regulation, starch, protein compensation, TF network, RNAi silencing, source-sink communication

## Abstract

Gluten proteins are responsible for the unique viscoelastic properties of wheat dough, but they also trigger the immune response in celiac disease patients. RNA interference (RNAi) wheat lines with strongly silenced gliadins were obtained to reduce the immunogenic response of wheat. The E82 line presents the highest reduction of gluten, but other grain proteins increased, maintaining a total nitrogen content comparable to that of the wild type. To better understand the regulatory mechanisms in response to gliadin silencing, we carried out a transcriptomic analysis of grain and leaf tissues of the E82 line during grain filling. A network of candidate transcription factors (TFs) that regulates the synthesis of the seed storage proteins (SSPs), α-amylase/trypsin inhibitors, lipid transfer proteins, serpins, and starch in the grain was obtained. Moreover, there were a high number of differentially expressed genes in the leaf of E82, where processes such as nutrient availability and transport were enriched. The source-sink communication between leaf and grain showed that many down-regulated genes were related to protease activity, amino acid and sugar metabolism, and their transport. In the leaf, specific proline transporters and lysine-histidine transporters were down- and up-regulated, respectively. Overall, the silencing of gliadins in the RNAi line is compensated mainly with lysine-rich globulins, which are not related to the proposed candidate network of TFs, suggesting that these proteins are regulated independently of the other SSPs. Results reported here can explain the protein compensation mechanisms and contribute to decipher the complex TF network operating during grain filling.

## Introduction

Wheat is one of the most important staple foods worldwide. It has unique viscoelastic properties for breadmaking due to its grain protein composition, allowing the production of many foods like bread and pasta (Shewry, 2009). However, wheat is also related to adverse reactions in humans, particularly proteins harboring epitopes that can trigger the immune response in celiac disease (CD), a chronic enteropathy mediated by human leukocyte antigen (HLA) HLA-DQ2 and HLA-DQ8 (Sollid, 2002). In addition to CD, there are other pathologies related to wheat consumption, such as Non-Celiac Wheat Sensitivity (NCWS), and wheat allergy, which includes baker’s asthma and Wheat Dependent Exercise Induced Anaphylaxis (WDEIA) (Aziz et al., 2016).

Gluten proteins are composed of monomeric and polymeric proteins, gliadins and glutenins, respectively. Gliadins are soluble in alcoholic solutions and are divided into three structural groups; ω-, α/β- and γ-gliadins based on their mobility in A-PAGE electrophoresis gels. Glutenins are the polymeric fraction, insoluble in alcoholic solutions and classified into High Molecular Weight (HMW) and Low Molecular Weight (LMW) glutenin subunits, based on their mobility in SDS-PAGE electrophoresis gels (Shewry and Halford, 2002).

The development of wheat varieties lacking the proteins responsible for triggering adverse reactions to wheat is an appealing target for Plant Biotechnology, and both RNA interference (RNAi) and CRISPR/Cas9 were applied, providing wheat lines with the CD-related gliadins strongly down-regulated or knock-out, respectively (Barro et al., 2016; Sánchez-León et al., 2018). In particular, RNAi technology was highly effective for the silencing of all three ω-, α/β-, and γ-gliadin fractions in wheat grain. However, the depletion of gliadin proteins affects the bread-making quality depending on the protein fraction being silenced (Gil-Humanes et al., 2012, 2014b). One wheat line denoted as E82 highlights among all RNAi lines produced, with a reduction of 98% of gluten content (Gil-Humanes et al., 2010), and the bread made using flour from this line was tolerated by NCWS patients (Haro et al., 2018), and did not elicit an immunogenic response in a blind randomized crossover gluten challenge with CD patients (Guzmán-López et al., 2021).

As a consequence of the down-regulation of gliadins by RNAi, this E82 line exhibits a marked readjustment in the grain proteins distribution in comparison to that of the wild type (WT); the gliadins were strongly decreased, the HMW glutenin subunits were not affected while the LMW glutenin subunits decrease, and the non-gluten proteins (NGPs), as globulins and triticins, increased, ensuring that the total nitrogen (N) content of the grain remains comparable to that of the WT (Gil-Humanes et al., 2010; Pistón et al., 2013; Ozuna et al., 2015; Barro et al., 2016). These protein compensation processes are particularly important to understand the molecular mechanisms operating in the grain, as they would allow these pathways to be targeted by using New Plant Breeding Techniques (NPBTs), such as CRISPR/Cas, for the development of new wheat varieties with specific nutritional profiles and unable to elicit an immunogenic response.

The accumulation of wheat prolamins during grain filling is mainly regulated at the transcriptional level by the interaction between the cis-regulatory elements (CREs), present in prolamin promoters, and the transcription factors (TFs) which recognize them (Kawakatsu and Takaiwa, 2010). Several CREs have been identified in the promoter sequences of seed storage protein (SSP) genes like gliadins and glutenins, being the endosperm box (E box), which is comprised of the prolamin box (P box) and the GCN4 motif, one of the most important (Kawakatsu and Takaiwa, 2010). The Storage Protein Activator (*TaSPA*), a wheat ortholog of basic leucine zipper (bZIP) opaque2 (*O2*) from maize, recognizes the GCN4 motif, while the DNA binding with one finger (DOF) Prolamin Binding Factor (*TaPBF*) recognizes the P box (Kawakatsu and Takaiwa, 2010). There are other well-conserved CREs and TFs involved in transcriptional regulation of SSPs genes, like the *TaGAMyB* TF that binds to the 5′-C/TAACAAA/C-3′-like motif, and the *TaFUSCA3* that binds to the RY motif (Kawakatsu and Takaiwa, 2010; Guo et al., 2015; Sun et al., 2017).

Apart from proteins, wheat grain is also an important source of carbohydrates, with starch accounting for ∼ 80% of its composition. Starch synthesis is catalyzed by enzymes like starch synthase (SS), and starch branching enzyme (SBE), among others (Shevkani et al., 2017). They are coded by starch synthesis-related genes (SSRGs) and they are regulated by TFs as bZIPs and NACs (Wang et al., 2013; Zhang et al., 2019). In addition, there are TFs actively involved in the regulation of both SSPs and SSRGs, like *TaSPA* and *TaPBF* in wheat, or *O2* and *PBF* in maize (Zhang et al., 2016; Guo et al., 2020; Orman-Ligeza et al., 2020).

During grain filling, active source-sink communication is a key point for the accumulation of SSPs and starch, and for therefore, a major determinant for wheat quality and grain yield. This source-sink communication controls the production and transport of photoassimilates from leaves to grains, and the capacity of grain to store them (Aguirrezábal et al., 2015; Smith et al., 2018). Moreover, around 70% of the N in wheat grain comes from senescing leaves, mainly in amino acid form, and it is used for the synthesis of SSPs during the development of the embryo (Peeters and Van Laere, 1994; Masclaux-Daubresse et al., 2008).

For the E82 line, with strongly down-regulated gliadins, a strong protein compensatory mechanism operates to maintain the grain N level. This line is an excellent material to understand the mechanisms of protein regulation in the grain, and how this compensatory phenomenon is driven by or affects other tissues such as leaves that act as the main suppliers of compounds that ultimately determine the nutritional profile of the grain. In the present work, we aimed to progress in deciphering the genetic and molecular mechanisms of protein compensation which operate in the grain of the wheat RNAi lines, to move forward in the development of new wheat varieties, safe for patients with gluten/wheat intolerance and possess exceptional nutritional properties.

## Materials and methods

### Plant material and experimental design

In this work, we used the E82 RNAi wheat line, derived from BW208, used as the WT (Gil-Humanes et al., 2010). The E82 line is the result of eight generations of self-pollination. This RNAi line contains the plasmids pGhp-ω/α and pghp8.1, harboring inverted repeat sequences of the most conserved regions from ω, α, and γ-gliadin genes, driven by endosperm-specific promoters (Pistón et al., 2008, 2009).

The two lines were grown in a greenhouse with a day/night regime of 12/12 h at 24/16°C. The seeds and flag leaf from three different plants as biological replicates were collected at 20 days after anthesis (DAA) for RNA extraction for each line and immediately frozen with liquid nitrogen and stored at –80°C. The seeds of three additional plants were also collected at harvest time for prolamin content determination.

### RNA extraction and sequencing

The grains and flag leaves of the WT and E82 at 20 DAA were milled, and the total RNA was extracted using the protocol of Direct-zol RNA Kits (zymoresearch.com). For grain tissue, we also performed the first step described in the protocol of Meng and Feldman (2010).

Total RNA was sent to Novogene (United Kingdom) Company Limited (25 Cambridge Science Park Milton Road, CB4 0FW, United Kingdom) for sequencing. The quality control of total RNA was performed by NanoDrop ND-1000 spectrophotometer (Thermo Fisher Scientific, Waltham, MA, United States) for preliminary quantitation and by 2% agarose gel electrophoresis for RNA integrity. The RNA integrity and quantification were also carried out by Novogene (Agilent 2100). The rRNA was removed using the Ribo-Zero kit. cDNA synthesis, library construction of 2 × 150 paired-end reads, and sequencing with Novaseq 6000 system (Illumina) were performed by Novogene.

### RNA-seq data analysis

Bioinformatic analysis was performed on a server with 64 cores and 128 GB of RAM with the operative system GNU/Linux Ubuntu version 18.04 (www.ubuntu.com, United Kingdom).

### Trimming

The trimming of raw reads and the adapters removing were performed with Trimmomatic-0.36 (params: ILLUMINACLIP:TruSeq2-PE.fa:2:30:10 AVGQUAL:20 LEADING:3 TRAILING:3 MINLEN:36) (Bolger et al., 2014). The ∼ 2% of reads per sample were discarded (Supplementary Table 1).

### Mapping

Reads were pseudoaligned by kallisto v0.44.0 (Bray et al., 2016) with default parameters (–pseudobam included) to the International Wheat Genome Sequencing Consortium (IWGSC) RefSeqv1.1 transcriptome annotation (Appels et al., 2018). The control quality of pseudoalignment was performed with samtools v1.3.1 (Li et al., 2009) (Supplementary Table 2). The summarized Transcript Per Million (TPM) matrix at gene level was generated with tximport v.1.14.2 (Soneson et al., 2016).

### Differential gene expression (DGE) analysis

DGE analysis was performed with edgeR v.3.22.5 (Robinson et al., 2009) in R v.3.6.3 (R Core Development Team, 2020). We established that differentially expressed (DE) genes had a False Discovery Rate (FDR) < 0.05. Two pair-wise comparisons were performed: WT grain tissue vs. E82 grain tissue, and WT leaf tissue vs. E82 leaf tissue. The genes with a sum of Counts Per Million (CPM) in all samples higher than 1 were considered in the DGE analysis. The complete expression data for all the genes and DGE analysis results are deposited in Gene Expression Omnibus (GEO) database with the identification number GSE199525.

### Gene Ontology (GO) and pathway enrichment analyzes

The GO and the Kyoto Encyclopedia of Genes and Genomes (KEGG) terms were obtained from RefSeq v1.1 annotation. We only use high confidence genes for the GO enrichment analysis and this was carried out with g:Profiler2 (Raudvere et al., 2019) for up- and down-regulated genes separately in each comparison. For pathway enrichment analysis we used also high-confidence genes.

### Seed-borne allergens and immune-responsive proteins

The annotation of genes coding for allergens and immunogenic proteins implicated in human diseases was obtained from Juhász et al. (2018). Prolamins genes from the work of Altenbach et al. (2020) and Huo et al. (2018a,b) were searched in the wheat genome to include them in the analysis.

### Gene Network Inference with Ensemble of trees (GENIE3) and Weighted Gene Co-expression Network Analysis (WGCNA) networks in bread wheat

Annotation for TF genes was obtained from Ramírez-González et al. (2018). The Gene Network Inference with Ensemble of trees (GENIE3) network (Ramírez-González et al., 2018) was used for predicting the downstream target genes of the DE TFs in our DGE analysis for WT vs. E82 comparison in the grain tissue. The top 1 million interactions of the GENIE3 network were used for the analysis, according to previous researches (Huang et al., 2018; Ramírez-González et al., 2018; Harrington et al., 2020). We calculated the shared Ratios of each DE TF gene (shared Ratio: number of DE target genes/number of target genes, for each TF gene) as described in Harrington et al. (2020). The DE TF genes with a shared Ratio > 0 were considered candidate TFs. The relationship between the candidate TF genes obtained from the GENIE3 network in grain tissue was visualized with igraph 1.2.4.2 (Csardi and Nepusz, 2006) and nodes (TF genes) were grouped into communities based on greedy optimization of modularity (Csardi and Nepusz, 2006; Murata, 2010). For those TF genes without annotation, the gene names of orthologs in *Arabidopsis thaliana* were included along with TFs gene ID.

To study the candidate TFs functions and their relationship with SSPs and SSRGs, we calculated the percentage of DE genes in the grain of E82 in each module of the Weighted Gene Co-expression Network Analysis (WGCNA) grain network, a co-expression network of 850 RNA-seq of wheat in different tissues and conditions (Ramírez-González et al., 2018). We repeated this process for the leaf tissue.

### Gene expression by quantitative real-time PCR (qPCR)

We selected DE genes to validate the results by qPCR. cDNA was synthesized using 1 μg of total RNA with qScript cDNA SuperMix (Quantabio, United States). qPCR was performed with SYBR Green Supermix (Bio-Rad, United States) on CFX Connect Real-time PCR Detection System (Bio-Rad). The Bio-Rad CFX Manager 3.1 was used for data analysis. For target efficiency, serial dilutions 1:2 were prepared for a bulk of grain and leaf samples separately. For data normalization, *CDC*, *RLI* and *ADP-RF* genes were used as reference genes (Giménez et al., 2011) using the primers listed in Supplementary Table 3.

### Prolamin quantification by Reverse Phase-High Performance Liquid Chromatography (RP-HPLC)

For prolamin extraction, 100 mg of flour from each line at 10, 20, 30, and 40 DAA were used per each biological replicate. The gliadin and glutenin were sequentially extracted following the protocol of Pistón et al. (2011), and protein quantification was performed by RP-HPLC (1200 Series Quaternary LC System liquid chromatography from Agilent Technologies) with a DAD UV-V detector at 210 nm. A 25 cm long column LiChrospher^®^ 100 RP8 (5 μm) (Merck) was used.

### Total protein, thousand kernel weight, and starch content

For total protein, grains of the WT and the E82 RNAi lines were collected at harvest and milled. Total protein content was calculated with Kjeldahl (%N x 5.7) method [standard ICC no. 105/2] for each biological replicate. The weight of thousand kernels was measured in triplicate. The starch content was determined with the standard ICC method no. 123/1 for each biological replicate. All these measures were expressed by the dry weight (DW) of flour.

### Proteomic data analysis

Grains of the WT and the E82 RNAi lines at harvest were collected and milled for proteomic data analysis. Protein digestion, liquid chromatographic and mass spectrometric analysis were performed as described in Vaquero et al. (2018) followed by custom Python scripts^1^ for counting the number of unique peptides assigned to each wheat protein fraction.

## Results

**FIGURE 1:**
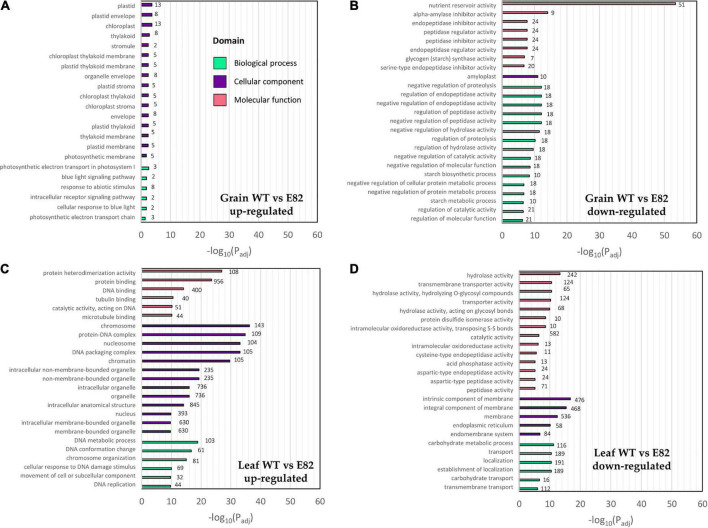
Gene Ontology (GO) enrichment analysis of up- and down-regulated genes in the wild type (WT) vs. E82 comparison in grain **(A,B)** and leaf **(C,D)** tissues. The top GOs are represented. Values on the bars represent the number of differentially expressed (DE) genes annotated with each GO term. *P*-value was adjusted with the Benjamini-Hochberg method.

We carried out a transcriptomic (RNA-seq) study for the E82 RNAi line and its WT (BW208) using grain and leaf samples at 20 DAA. Trimmed reads were pseudoaligned with Kallisto to the RefSeq v1.1 transcriptome annotation, obtaining an average of 71.54% of clean reads mapped (Supplementary Table 4). Two pair-wise comparisons were performed for the DGE analysis: WT vs. E82 for grain and leaf tissues separately. The WT vs. E82 grain pair-wise comparison provided 109 genes up-regulated in E82 compared to the WT, while 859 genes were down-regulated. In contrast, the WT vs. E82 leaf pair-wise comparison provided 4,230 and 1,593 genes up-and down-regulated, respectively. For grain, the most enriched GO terms for the up-regulated genes in E82 were related to the Cellular component domain, some of them specifically in the chloroplast location, and the Biological Process domain, with genes related to response to abiotic stimulus (Figure 1A). While GO terms related to nutrient reservoir activity and starch metabolism were enriched in GO enrichment analysis for down-regulated genes in the E82 line (Figure 1B).

### Grain proteins are strongly affected by the RNAi

**FIGURE 2:**
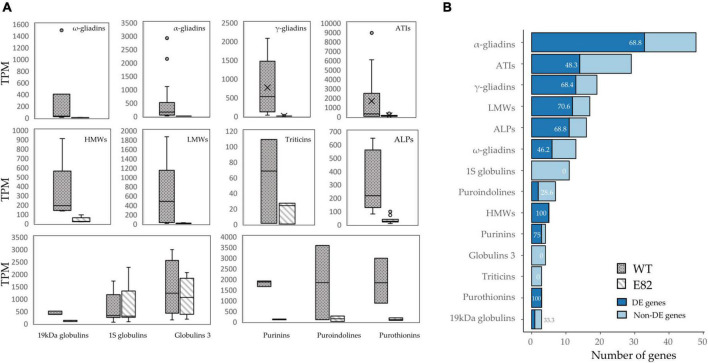
**(A)** Overview of the expression of differentially expressed (DE) genes coding for grain proteins between the wild type (WT) and E82. For globulins and triticins, we represented all genes. The line in the box represents the median value. **(B)** Number of DE and non-DE genes for each protein type. The number in bars indicates the percentage of DE genes. Gene annotations were extracted from the RefSeq v1.1, Juhász et al. (2018) and Altenbach et al. (2020) and Huo et al. (2018a,b), and for triticins, we performed a blastp of three protein sequences of triticins from Butte 86 summarized in Altenbach et al. (2020) against the wheat genome.

The expression of gliadin genes, targeted by the RNAi silencing fragment, was strongly decreased in the E82 line at 20 DAA, but glutenin genes and other non-targeted grain proteins like avenin-like proteins (ALPs), α-amylase/trypsin inhibitors (ATIs), puroindolines, purinins, and purothionins, had lower expression in E82, which correspond with a high number of DE genes in WT vs. E82 grain comparison (Figure 2). Interestingly, globulins and triticins genes were not DE in the E82 RNAi line (Figure 2), except for one gene encoding 19 kDa globulin.

The synthesis and deposition of gliadins and glutenins were also monitored by RP-HPLC throughout grain filling. As shown in Supplementary Figure 1, the E82 line had lower content than the WT for the α- and γ-gliadins from the first stages of grain filling, including 20 DAA, while the ω-gliadins content was lower in E82 only at 40 DAA (Supplementary Figure 1A). In the case of glutenin proteins, the HMW content was higher in E82 than the WT in the first stages (Supplementary Figure 1A) while the LMW content was lower in E82 at 20 DAA (Supplementary Figure 1A).

The total protein content for the different gliadin fractions obtained by RP-HPLC (Supplementary Figure 1B) and the abundance of unique peptides by LC-MS/MS (Supplementary Table 5) at harvesting confirmed that E82 has less content of gliadins and a lower abundance of unique peptides for all the gliadin fractions than the WT (Supplementary Figure 1B and Supplementary Table 5). The content of glutenin protein fractions and their abundance of unique peptides varied in the same way, decreasing only for the LMW in the E82 RNAi line (Supplementary Figure 1B and Supplementary Table 5). Moreover, the NGPs that were increased in E82 at harvesting (Supplementary Figure 1C), particularly globulins and triticins, lipid transfer proteins (LTPs), and serpins, also increased the abundance of unique peptides (Supplementary Table 5). All these protein rearrangements do not affect the total grain N content, which was not statistically different between E82 and the WT (Supplementary Figure 1C).

### Starch synthesis-related genes are also affected in the RNAi line

**FIGURE 3:**
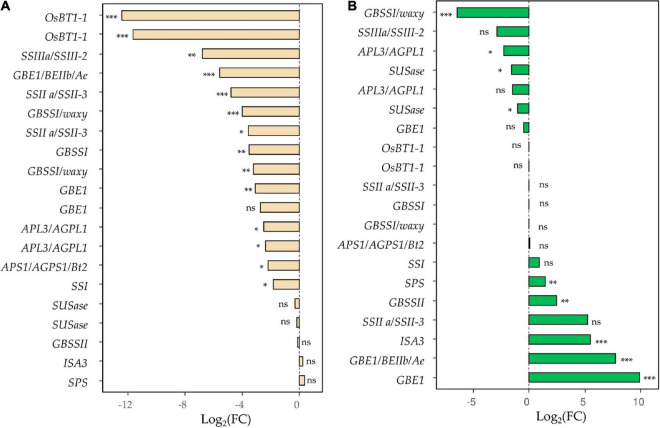
Starch synthesis-related genes (SSRGs) differentially expressed (DE) in **(A)** grain and/or **(B)** leaf tissue for the pair-wise comparison wild type (WT) vs. E82. *, FDR < 0.05; **, FDR < 0.01; ***, FDR < 0.001; ns, non-significant; FDR, False Discovery Rate.

RNAi silencing in the E82 line also affects the starch content and the genes involved in its regulation. For grain tissue, 14 SSRGs, coding for key enzymes in starch synthesis, were down-regulated compared to the WT at 20 DAA (Supplementary Table 6 and Figure 3), which agrees with the lower content of starch and the lower kernel weight detected in the E82 line at harvesting (Supplementary Figure 1D,E), and with the lower normalized gene expression of SSIIIa/SSIII-2 genes in the grain of E82 obtained by qPCR (Supplementary Figure 2). Moreover, for the flag leaf, the expression of five and four SSRGs were up-and down-regulated, respectively, in the E82 line (Supplementary Table 6 and Figure 3).

### Candidate TFs for the grain protein and starch synthesis regulation in the E82 RNAi line

Many of the genes proposed in previous research for the regulation of SSPs and starch synthesis are listed in Supplementary Table 7, including the *TabZIPs* which play a role in the amylose biosynthesis in wheat (Kumar et al., 2018). Among these genes, transcriptomic data showed that the A-subgenome homeolog of the *TaPBF* was significantly down-regulated in the E82 line, while the other two homeologs present a low, not significant expression (Supplementary Table 7). Moreover, in the qPCR analysis, the pooled three homeologs for *TaPBF* were down-regulated in the E82 line (Supplementary Figure 2). However, the rest of the TFs listed in Supplementary Table 7 were not DE in the grain of E82, and therefore they may not be relevant for explaining the rearrangements in the regulation of the SSP and starch synthesis genes in the E82 line. The expression of homeologs of the *TaSPA*, which has a key role in the biosynthesis of prolamins and starch (Guo et al., 2020), was not over the threshold to be included in the DGE analysis.

**FIGURE 4:**
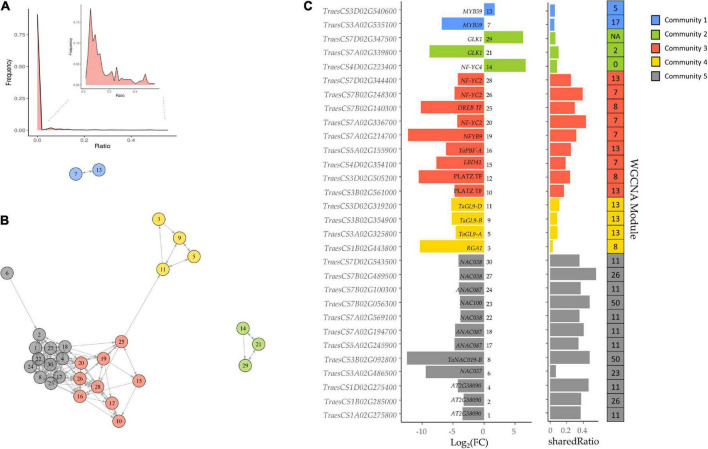
**(A)** Frequency of shared genes between Gene Network Inference with Ensemble of trees (GENIE3) transcription factors (TFs) target genes and differentially expressed (DE) genes in the grain of E82 compared to the wild type (WT). For improving the representation of frequencies, we remove those TFs with zero shared genes in the insert. **(B)** Network of candidate TFs implicated in the protein and starch changes in the grain of E82. All these genes are DE TF with a shared Ratio > 0. The different colors represent the community of nodes detected based on greedy optimization of modularity. **(C)** Fold-change (FC) values and shared Ratio of each gene in the network. Gene name (*A. thaliana* ortholog information) and network ID are represented in the plot. On the right, there is the module of the Weighted Gene Co-expression Network Analysis (WGCNA) network for grain tissue to which each TF belongs.

Because of the above results, the GENIE3 network was used to find other TFs related to the SSPs and starch synthesis. This network provides biologically-relevant information based on TF’s targets prediction, and it was demonstrated for bread wheat TFs related to senescence (Harrington et al., 2020). We searched the DE TFs in the grain of E82 in the wheat GENIE3 network, selecting those which had shared Ratio values higher than zero (at least one of their target genes was DE in the grain of E82). Only 30 out of 3,000 TFs present in the GENIE3 network met both criteria (Figure 4), being clustered in five communities (Figures 4B,C). The TF *TaPBF-A* belongs to community 3, which together with community 5, has the highest shared Ratio values (Figure 4C). Therefore, these TFs form a complex regulatory cascade and will be considered candidate TFs throughout this work.

To establish the putative function of each TF, we summarized (Supplementary Table 8) the top three GO terms most enriched for each TF from previously GO enrichment analysis for the GENIE3 network. Many of the TFs were in communities 3, 4, and 5, and associated with carbohydrate-mediated signaling, detection of nutrients, glycogen metabolism, lipid storage, regulation of endopeptidase activity, regulation of catalytic activity, energy reserve metabolic process, and seed oil body biogenesis. TFs in communities 2 and 1 were related to photosynthesis and defense response processes, respectively (Supplementary Table 8).

**FIGURE 5:**
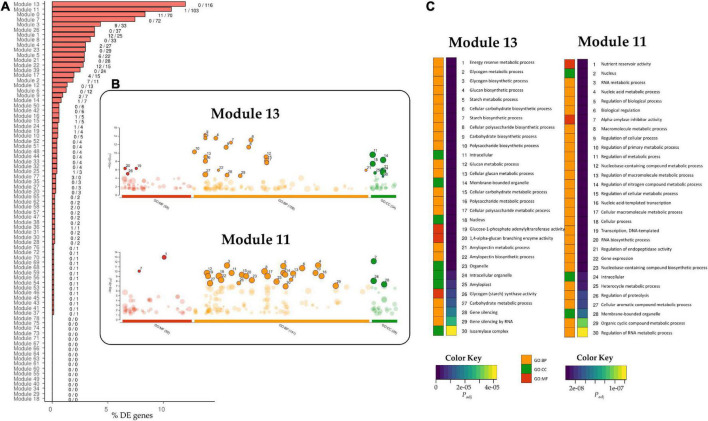
**(A)** Percentage of differentially expressed (DE) genes that belong to each module in the Weighted Gene Co-expression Network Analysis (WGCNA) network for grain tissue. The label close to the bars indicates the number of up/down-regulated genes in each module. **(B)** Manhattan plot of the Gene Ontology (GO) enrichment analysis of genes in modules 13 and 11 of the WGCNA network from grain tissue. The number of enriched GOs per domain is indicated between parentheses. The enriched GOs are represented, and the top 30 GO enriched are highlighted. **(C)** The adjusted *P*-value with the Benjamini-Hochberg method of the top 30 enriched GOs is represented per domain in modules 13 and 11. GO:MF, Molecular Function domain; GO:BP, Biological Process domain; GO:CC, Cellular Component domain.

Each candidate TF was then assigned to a module according to the grain co-expression network constructed with WGCNA (Figure 4C), and the percentage of DE genes in each module for the E82 grain was represented (Figure 5A). The *TaPBF-A* was addressed to module 13 (Figure 4C), together with a high number of other down-regulated genes in E82 (Figure 5A). Next, we extracted all the genes from this module and performed GO enrichment analysis; the GO terms related to carbohydrate metabolism were enriched (Figures 5B,C), and GOs related to amino acid metabolism stand out (Supplementary Table 9). In addition, module 11 was also overrepresented among the candidate TFs and has a high percentage of DE genes in the grain of E82 (Figures 4C, 5A). As for module 13, we performed GO enrichment analysis of genes included in module 11, and we found nutrient reservoir activity and α-amylase inhibitor activity terms enriched (Figures 5B,C).

**FIGURE 6:**
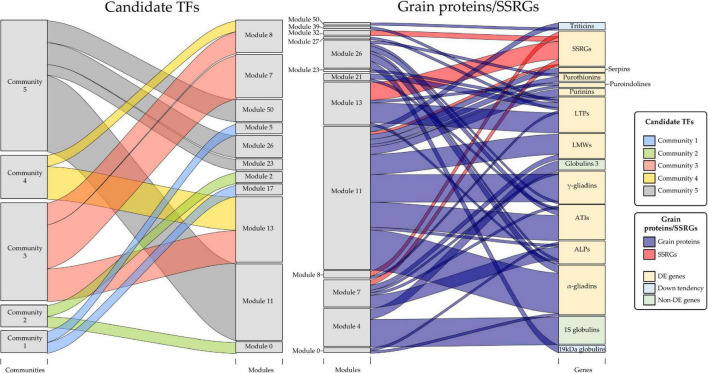
Relationships between communities of candidate transcription factors (TFs) and modules of Weighted Gene Co-expression Network Analysis (WGCNA) grain co-expression network (left), and between these modules and grain proteins/starch synthesis-related genes (SSRGs) (right). The size of the gray boxes indicates the number of genes contained in each category. All the grain protein genes and SSRGs used for representation were differentially expressed (DE) in our RNA-seq analysis. In the case of grain protein genes, the globulin ones non-DE were included for discussion purposes. Down tendency: genes with non-significant lower expression in E82, except for one DE gene encoding 19 kDa globulin.

The next step was to establish the match between the modules where the candidate TF genes were and the modules in which the grain protein genes and SSRGs were included. Other grain proteins such as LTPs and serpins, in the list of allergens and immune-responsive proteins implicated in human diseases listed in Juhász et al. (2018), were added to the study. We also searched these genes in the WGCNA grain co-expression network (Figure 6). Briefly, many gliadin and glutenin DE genes in the grain of E82 shared the same modules as the candidate TFs (Figure 6). The same occurs with other grain protein genes such as ATIs, ALPs, purothionins, puroindolines, purinins, serpins, and LTPs (Figure 6). Some grain protein genes such as globulins, including triticins genes, were not DE in our RNA-seq data analysis, although some of them showed a slight tendency to decrease in E82, being one 19 kDa globulin gene down-regulated (Figures 2A,B). While the genes for globulin 1S and globulin 3 were included in module 4 (not containing any of the candidate TFs), the 19 kDa globulin and triticin genes were included in module 26, and modules 11 and 50 respectively, with candidate TFs of community 5 (Figure 6). On the other hand, all the SSRGs down-regulated in E82 grain were also included in the same modules as the candidate TFs, except for *SSIIa/SSII-3* genes (Figure 6).

### The source-sink communication during grain filling is greatly affected by RNAi silencing

**FIGURE 7:**
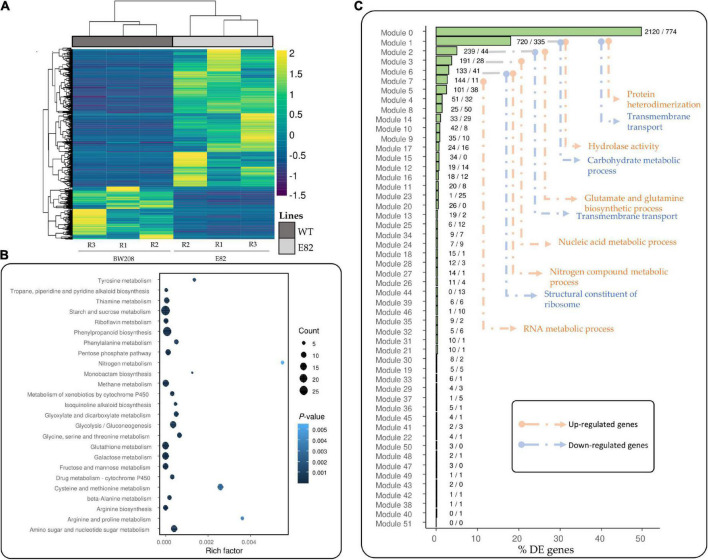
**(A)** Heatmaps of Transcript Per Million (TPM) for differentially expressed (DE) genes for the wild type (WT) vs. E82 comparison in leaf tissue. Values are scaled by row (–1.5 to 2). Dendrograms of genes and lines were represented. **(B)** Kyoto Encyclopedia of Genes and Genomes (KEGG) pathway enrichment analysis for down-regulated genes in E82 leaf tissue against the WT. The rich factor is the division between the number of DE genes annotated and the total number of genes annotated in each pathway. The count value is the number of DE genes annotated in this Kegg Orthology (KO). **(C)** Percentage of DE genes which belong to each module in the Weighted Gene Co-expression Network Analysis (WGCNA) network for leaf tissue. The label close to the bars indicates the number of up-/down-regulated genes in each module. The most enriched GOs for the set of up- and down-regulated genes in each module were represented following the arrows. R: replicate.

We also carried out an RNA-Seq analysis in leaves to determine if the transcriptomic changes found in the grain of the E82 line during grain filling could modulate the transcriptome of the leaf. As shown in Figure 7, there were many transcriptomic changes in the leaf of E82 against the WT. In fact, there were more DE genes in the E82 leaf than in the grain, and a higher number were up- than down-regulated. Many of the up-regulated genes were related to the DNA metabolism and chromosome organization (Figure 1C), while the down-regulated ones were related to peptidase activity, carbohydrate metabolism, and transport (Figure 1D, Supplementary Table 6 and Figure 3B). Moreover, starch and sucrose metabolism and amino acids metabolism pathways were enriched in the down-regulated genes of E82 (Figure 7B). All these processes are linked to source-sink interactions during grain-filling (Yu et al., 2015). As for grain tissue, the WGCNA co-expression modules ordered by their percentage of DE genes were represented (Figure 7C). The higest percentages of DE genes in the leaf were in modules 0, 1, 2, 3, 6, and 7 (Figure 7C). Then, a GO enrichment analysis was performed for the up-and down-regulated genes in the modules with a higher percentage of DE genes: protein heterodimerization, hydrolase activity, and glutamate and glutamine biosynthesis processes, among others, were enriched; GOs as transmembrane transport and carbohydrate metabolism were only enriched in the down-regulated genes (Figure 7C).

Then, we decided to study more in detail the genes related to peptidase activity and nutrients transport in the leaf of E82 because of their implication in the source-sink communication and, in the end, in the SSP and starch content in grain.

#### Proteases and protease inhibitors in the leaf of E82

The Papain-like cysteine proteases (CysProt), belonging to C1A according to the MEROPS database (Rawlings et al., 2016), play a main role in the proteolytic activity and nutrient remobilization during leaf senescence. Then, genes annotated in RefSeq v1.1 with the PFAM “Peptidase_C1” (PF00112) were searched on WheatMine website (https://urgi.versailles.inra.fr/WheatMine). Among them, one ortholog of *HvPap1* of *Hordeum vulgare* (NCBI Acc. N.: BN000093), was down-regulated in E82 (Supplementary Table 10). In addition, four and five (*HvPap4* orthologs, RD19D, *Psy1-D1* ortholog, and *HvPap14* ortholog) CysProt genes were down- and up-regulated in E82, respectively (Supplementary Table 10). In the case of cystatins, specific modulators of C1A peptidase activities, we looked for the cystatin domain (IPR000010) in WheatMine. There were five DE cystatin genes in E82. One of them was up-regulated in E82 (ortholog of *Icy2* of *H. vulgare*) (Supplementary Table 10). The serine proteases, together with CysProt, are also the most abundant enzymes associated with leaf senescence (Roberts et al., 2012). In the case of these peptidases, we filter genes with serine-type peptidase and endopeptidase activity in RefSeq v1.1. Briefly, 20 of these genes were up-regulated and 15 were down-regulated (Supplementary Table 11).

#### Sugar and amino acid transporters in the leaf and grain of E82

**FIGURE 8:**
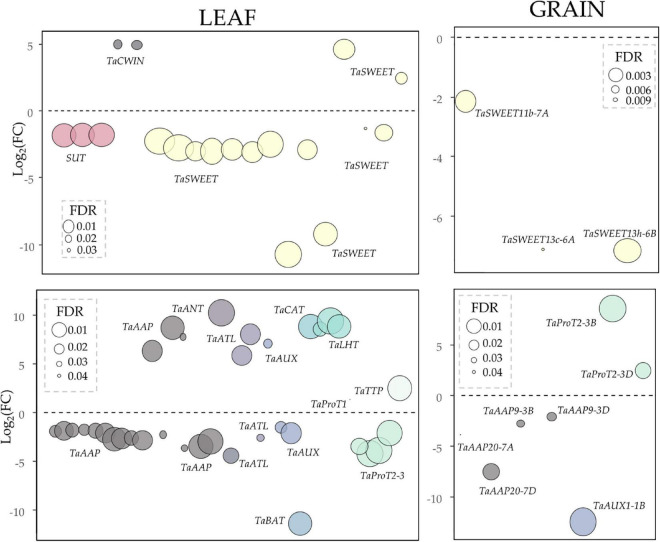
Sugar transporters (up) and amino acid transporters family (ATF) (down) genes differentially expressed (DE) in the leaf and grain of E82. The Log_2_(FC) is represented, those genes with Log_2_(FC) > 0 are up-regulated in E82, and those with Log_2_(FC) are down-regulated. The size of the dots corresponds to the FDR of each gene. FC, fold-change; FDR, False Discovery Rate.

Both, carbohydrate and transmembrane transport GOs were enriched among the down-regulated genes in the leaf of E82 (Figure 1D). The carbohydrate transport genes were identified accordingly to the work of Gautam et al. (2019), and the DE ones are listed in Supplementary Table 12. For carbohydrate transport, many of the *TaSWEET* and *SUT* genes were down-regulated in the leaf of E82 (Figure 8 and Supplementary Table 12). In addition, the expression of *TaSWEET13* homeolog genes (*TraesCS6B02G421800* and *TraesCS6D02G367400*) was also studied by qPCR, confirming the down-regulation in the leaf of E82 against the WT (Supplementary Figure 2). While in the grain, only three *TaSWEET* genes were down-regulated in E82 (Figure 8).

Concerning amino acid transporters, they have been identified accordingly to the work of Tian et al. (2020), and the DE genes were listed in Supplementary Table 12. Many of the amino acids permease (AAP) genes, which transport a wide range of amino acids (Kohl et al., 2012), were down-regulated in the leaf of E82, except for three of them which were up-regulated: *TaAAP22-7A*, *TaAAP6-2A*, and *TaAAP8-3A* (Figure 8 and Supplementary Table 12). Four genes encoding proline transporters (ProTs) 2 and 3, proline-specific transport, were down-regulated in the E82 leaf and one encoding ProT1 was slightly up-regulated, while two of them were up-regulated in the E82 grain (Figure 8 and Supplementary Table 12). Interestingly, three genes encoding lysine-histidine-like transporters (LHTs), associated with specific lysine-histidine transport, were highly up-regulated in the E82 leaf (Figure 8 and Supplementary Table 12).

## Discussion

The down-regulation of wheat gliadins by RNAi provided lines with a reduction of up to 98% of gluten in the grain (Gil-Humanes et al., 2010). The most outstanding line, named E82, showed the lowest amount of CD immunogenic epitopes (Sánchez-León et al., 2019), and elicited no immunogenic response in celiac disease patients after the oral consumption of E82 bread (Guzmán-López et al., 2021). Moreover, this line presents sensory properties comparable to that of the WT flour, suggesting that gliadins could not play a relevant role in texture, aroma, flavor and appearance as much as other grain components (Cho and Peterson, 2010; Gil-Humanes et al., 2014a). A common feature of this and other RNAi lines with the SSPs silenced was the rearrangement of the grain proteins to provide total N contents comparable to the WT (Gil-Humanes et al., 2012; Altenbach et al., 2019; García-Molina et al., 2019), which indicates that a compensatory mechanism is operating. However, depending on the SSP being targeted, there are different profiles of protein compensation. For example, line D793, with all the gliadin fractions down-regulated, shows a higher content of HMW glutenin subunits and lower LMW content than the WT (Gil-Humanes et al., 2011; Marín-Sanz et al., 2022). In contrast, when only the γ-gliadins are targeted, RNAi lines present higher content of both ω- and α-gliadins (Pistón et al., 2011). In RNAi lines for silencing the ω-5 gliadins, an increase in NGPs, HMW and LMW glutenin subunits, and α-gliadins were reported (Altenbach et al., 2014). However, if ω-1,2 gliadins were silenced, all gliadins and LMW glutenin subunits were also suppressed, and major compensation with HMW glutenin subunits and NGPs occurred (Altenbach et al., 2019). The protein compensation was also reported in maize RNAi lines, in which the reduction of the 22-kDa α-zeins was compensated by the 19-kDa α-zeins, and vice versa (Huang et al., 2004), and in rice mutants, in which the low glutelin content was compensated by other SSPs such as prolamins (Iida et al., 1993). In the case of the E82 line, with all three ω-, α- and γ-gliadin fractions down-regulated, an increase in the NGPs and a reduction in LMW were reported, leading to the decrease in the content of total glutenins (Pistón et al., 2013; Barro et al., 2016). The reduction of LMW could be due to an off-target silencing because of the high homology between the LMW mRNA sequence and the small interfering RNA (siRNA) formed from the hairpin RNA (hpRNA) (Gil-Humanes et al., 2014b). Altogether, it is clear that different compensatory mechanisms are at work in response to the depletion of the different protein fractions by RNAi. Moreover, CRISPR/Cas targeted mutations seem to provide a similar response in the wheat grain (Sánchez-León et al., 2018). Understanding the molecular mechanisms underlying this process of protein compensation in the grain will allow us to make fine adjustments, using CRISPR/Cas techniques, toward developing wheat lines with improved nutritional properties and suitable for people who suffer from any wheat-related pathology.

In this work, the transcriptome of the line E82 in both grain and leaf during grain filling provides new insights into both protein rearrangements and grain-to-leaf communication pathways when all gliadins are strongly silenced. A very high number of genes were DE in the leaf of E82, comprising processes such as nutrient availability and nutrient transport, while in the grain, many genes related to the protein synthesis, particularly genes encoding gluten proteins, along with ALPs, ATIs and LTPs, and genes related to the starch synthesis, were down-regulated, in concordance with the grain protein composition and the slight reduction of the starch content found in the grain of E82. Among the well-known TFs involved in prolamin and SSRGs regulation, the DOF-type *TaPBF-A* which binds to the P-box motif of prolamins genes (Mena et al., 1998; Zhang et al., 2016; Guo et al., 2020), was down-regulated in E82. Despite the relation between *TaPBF* and the SSRGs has to be further studied, some genes implicated in the starch synthesis also present in their promoters the *TaPBF* binding motifs (P-box and Pyrimidine box), and they can be targets of this TF, as predicted by the GENIE3 network (Orman-Ligeza et al., 2020), and in line with our transcriptomic results. Surprisingly, the rest of the well-known TFs related to the regulation of the synthesis of prolamins and starch were not DE in the grain of E82, which prompted us to expand the study to all the TFs to elucidate their role in the grain composition of the E82 line.

Among all the TFs, we selected those that were DE in E82, obtaining a putative TF regulatory cascade for the proteins and starch synthesis in the grain. This network comprised five communities, being three of them closely linked to each other, and the *TaPBF-A* gene included in one of them (community 3). The relationship between *TaPBF-A* and the regulation of prolamins, and some of the starch synthesis enzymes, suggests that not only this TF could be responsible for the shifts in the protein fractions observed in the grain of E82, but also those TFs closely related in the network would be potential candidates to regulate the grain protein genes and the SSRGs expression. Specifically, *TaNAC019* binds to the promoters of *TaGlu-1* loci on the homeologs chromosomes 1A, 1B, and 1D, which encode the HMW glutenin subunits. The *TaNAC019-B* homeolog was also present in our candidate TFs network in the community containing NACs, and preceding the *TaPBF-A* one. Recently, the role of this gene in the synthesis of SSPs and starch has been determined by using mutants for *TaNAC019* in wheat (Gao et al., 2021). The searching of the candidate TFs, protein genes and SSRGs in the co-expression modules of the WGCNA network, provided that many of the DE prolamin genes were classified in the same modules as the candidate TFs in the two largest communities in our network, which include *TaPBF-A* and *TaNAC019-B*. In addition, the DE genes that encode for ATIs, ALPs, purinins, purothionins, puroindolines, serpins, and LTPs were also classified in the same co-expression modules as the candidate TFs, suggesting that all these grain proteins may be subjected to the same regulatory network. However, the genes annotated as ω-gliadins and HMW were not considered as they are not present in the published WGCNA network for wheat, probably because the network was proposed before these genes were included in the wheat genome annotation. Except for one gene encoding 19 kDa globulin, globulin genes did not change their expression significantly in the grain of E82, but participated in the protein compensation of this and other RNAi lines (Gil-Humanes et al., 2011; Barro et al., 2016), suggesting that globulins would be regulated separately to prolamins and other grain proteins. In fact, globulins 3 and 1S globulins were not included in the same modules as the candidate TFs. Interestingly, an increase in the lysine-rich proteins (globulins), in addition to a high content of free lysine, was obtained in a triple null wheat line for the *TaPBF* gene homeologs (Moehs et al., 2019), suggesting that no relationship between the *TaPBF* and the increasing of globulins content exists. Moreover, the above triple null mutant presented lower content of gliadins and LMW, which agrees with the E82 profile. In addition, this mutant also showed a lower content of free proline and glutamine in the grain compared to its WT, as expected because of the role of this TF in the prolamins regulation (Moehs et al., 2019). Similarly, the *lys3a*, a previous mutant of barley with a missense allele in a domain of the PBF, showed a reduction in hordein content, while free and protein-bound lysine increased (Shewry et al., 1980; Moehs et al., 2019). Overall, it seems that most of the globulin genes are not the targets of the *TaPBF*, and their increase in the E82 line could be a consequence of a different regulation pathway and/or they are favored by the free amino acids profile lead after the down-regulation of gliadins.

Recently, a list of candidate TFs to regulate the SSPs was proposed for *Triticum urartu* by using a gene co-expression analysis (Luo et al., 2021). Our results agree with those, as eight orthologs TFs of bread wheat were in community 5 (NAC community) and five in community 3 (*TaPBF-A* community), so they are potential candidates for the regulation of the SSPs in bread wheat. Interestingly, in our NAC community, one of the TF (*TraesCS3A02G486500*) which encodes the NAC057 ortholog of *A. thaliana* and has been identified as NAM-like protein (Guerin et al., 2019), was not listed as a putative regulator for SSPs by Luo et al. (2021). This gene is not the target of any DE TFs in the grain of E82 and its function in wheat is still unknown. However, considering its position in the candidate TFs regulatory network, it could have a prominent role in the regulation of the SSPs. In addition to the communities containing *TaPBF-A* and *TaNAC019-B*, there is a third community (community 4) connected to them by *TraesCS7B02G140300*, which encodes a TF member of the DREB subfamily A-5 of ERF/AP2 family (Ramírez-González et al., 2018). In this community, the sequence of three of the four TFs found (*TraesCS3A02G325800*, *TraesCS3B02G354900* and *TraesCS3D02G319200*) presents a high similarity with the TF *TaGL9* (GLABRA2-like clone 9) from *Triticum aestivum*. This *TaGL9* contains a putative lipid-binding domain and it could be involved in the regulation of lipid biosynthesis and their transport (Kovalchuk et al., 2012). In fact, the *TaGL9* genes have the same expression pattern as genes encoding LTPs, suggesting that either the LTP genes are regulated by *TaGL9* or both genes are regulated by other TFs (Boutrot et al., 2007; Kovalchuk et al., 2012; Chew et al., 2013). Our results agree with both, as some of the LTP genes were down-regulated in the grain of E82, as well as all three *TaGL9* homeologs, being most of them located in the same co-expression module (module 13). The connection of *TaGL9* with *TaPBF-A* and *TaNAC019-B* described above suggests a common regulation of SSPs, starch, and LTPs.

We also focused on the leaf transcriptome of E82 during the grain filling as the source-sink communication is important for plant productivity, enhanced by source activity and sink strength. Source activity comprises the photosynthesis or nutrient remobilization rates, and sink strength is the capacity to import photoassimilates or biosynthetic rate of starch and proteins among others (Yu et al., 2015). However, not much information on the regulation of such source-sink communication during grain filling is available (Yu et al., 2015). Concerning the leaf transcriptome of E82, we have obtained many DE genes compared to the WT, many of them related to amino acids transport, carbohydrate transport, amino acids and sugar metabolisms, and peptidase activity. In this respect, carbohydrates are the main photoassimilate produced in the leaves. The leaf of E82 is also affected in the production of carbohydrates as many genes related to the carbohydrate metabolism process were down-regulated, and this may lead to lower availability of this nutrient to be transported to filling grain. This may explain that carbohydrate transporters were mainly down-regulated in the leaf of E82, contributing to the lower starch content found in the grain of E82 at harvest in comparison to that of the WT.

Protein degradation allows the recycling of N and other nutrients during leaf senescence (Bhalerao et al., 2003; Guo et al., 2004). In the line E82, we found many DE genes among the C1A CysProt and the serine-type peptidases, two of the most abundant protease families in plants and important during leaf senescence for the remobilization of nutrients from source tissues to the storage ones (Roberts et al., 2012; Rawlings and Salvesen, 2013; Díaz-Mendoza et al., 2014; Liu et al., 2018). For example, the orthologs in wheat of the *HvPap14*, *HvPap4*, and RD19D genes in the leaf of E82 were up-regulated, suggesting that those proteases may actively provide substrates for nutrient remobilization to the grain of E82. Regarding amino acids, the synthesis of some of them was enriched among the down-regulated genes in the RNAi line, which may indicate a lower availability. As we hypothesized in previous works, the protein compensation studied in RNAi lines is a selective process that tries to prioritize the compensation of proteins with similar amino acid profiles to the silenced proteins over the least similar ones, as the protein compensation is closely linked to the availability of amino acids (Pistón et al., 2013). As mentioned before, we observed that the γ-gliadins reduced lines presented an increase of the rest of the prolamin proteins with similar amino acid profiles (Pistón et al., 2011). However, the lines with a reduction of all the gliadins and the LMW presented an increase in HMW and NGPs, including albumins and globulins, which present different amino acid compositions (Pistón et al., 2013). Among the wheat protein fractions, globulins present the highest lysine content and the lowest proline content, while prolamins are rich in proline and glutamine, and have the lowest content of lysine and other essential amino acids such as histidine (Shewry et al., 2009). Accordingly, genes involved in the metabolism of proline were down-regulated in the leaf of E82, in line with the fact that the grain would require less content of this amino acid. Moreover, regarding ProTs, which have a high affinity for proline and play a key role in the rapid distribution of this amino acid (Ueda et al., 2008), *TaProTs 2-3* were down-regulated in the leaf of E82, which could be a consequence of the down-regulation of proline metabolism in the leaf. Regarding lysine-rich proteins, three genes encoding LHTs were strongly up-regulated in the leaf of the RNAi line, which could contribute to the high content of total lysine and lysine-rich proteins in the grain of E82 as reported previously (Gil-Humanes et al., 2014a; Barro et al., 2016). In addition to ProTs and LHTs, a large number of genes encoding AAPs were DE in the leaf of E82, being the expression of AAPs regulated in the sink-source communication (Kohl et al., 2012). The above results suggest that the amino acids transporters could play an important role in the E82 grain protein compensation, however, further analyzes are needed to study their implication in this process.

**FIGURE 9:**
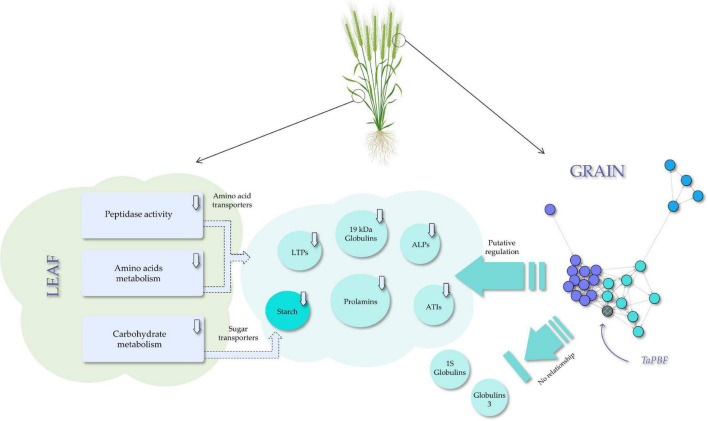
Model of the transcription factors (TFs) network role in the regulation of the seed storage proteins (SSPs) and starch synthesis, and the effect on the grain-leaf communication-related processes in a RNAi line with gliadins down-regulated.

Taking together, the results reported here show that gliadin silencing in wheat has a great transcriptomic effect on the leaves than on the grain. However, the changes observed in the leaf transcriptome are probably a consequence of the changes observed in the grain during grain filling. Therefore, no changes in the leaves are expected before or after this physiological process. These results provide new insights to decipher the complex regulation network which operates during grain filling in response to the down-regulation of prolamins by RNAi: as a summary, the network of TFs in the grain could regulate the starch and SSPs synthesis, but some globulins would be exempt from this regulation, having the principal role in protein compensation (Figure 9). This transcriptomic information also highlights the grain-leaf communication (Figure 9) and could be used to redesign the wheat grain protein composition and control the nutrient supply chain during grain filling. All these objectives could be now achievable using CRISPR/Cas technology, enabling the development of new healthier wheat varieties, totally safe for those suffering adverse reactions to wheat.

## Data availability statement

The datasets presented in this study can be found in online repositories. The names of the repository/repositories and accession number(s) can be found below: https://www.ncbi.nlm.nih.gov/, PRJNA820621; https://www.ncbi.nlm.nih.gov/, GSE199525.

## Author contributions

FB conceptualized the project. FB and MM-S designed the experiment and wrote the manuscript draft. MM-S performed the specific experiments and data analysis. Both authors have read and approved the final manuscript.

## Footnotes

1
https://github.com/MiriamMarinS/ProtEp
