# Supplementary material for: RNAi silencing of wheat gliadins alters the network of transcription factors that regulate the synthesis of seed storage proteins toward maintaining grain protein levels
Source: Front Plant Sci. 2022 Aug 8;13:935851. doi: 10.3389/fpls.2022.935851 (PMC9395171; doi:10.3389/fpls.2022.935851)
Supplement: Supplementary file 1 [file Table_1.docx]

Supplementary Material

# Supplementary Figures


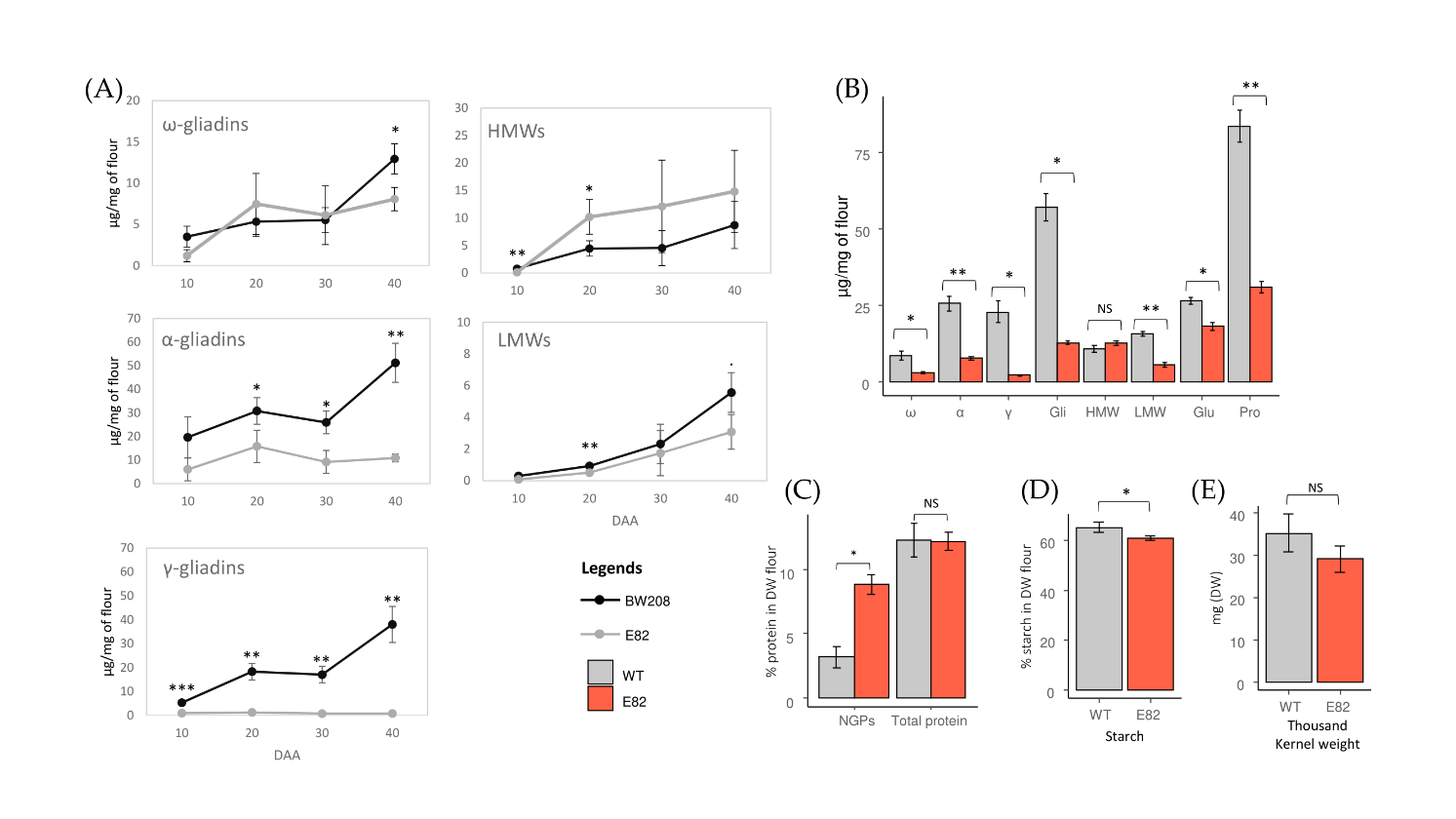


**Supplementary Figure 1.** (A) Content of gliadins and glutenins of the wild type (WT) and E82 throughout grain development. Bars indicate standard deviation. ANOVA was performed for each DAA between BW208 and E82. (B) Comparative gliadin and glutenin grain protein content at harvest between the WT and E82, (C) non-gluten proteins (NGP) and total grain protein contents at harvest between the WT and E82, (D) Starch content in grain tissue at harvest for BW208 and E82. (E) Thousand kernel weight for the WT and E82. Bars indicate the standard error of three biological replicates. ·, *P* ~ 0.05; *, *P* < 0.05; **, *P* < 0.01; ***, *P* < 0.001; NS, non-significant. Gli: total gliadins; Glu: total glutenins; Pro: total prolamins; DW: dry weight; DAA: days after anthesis.


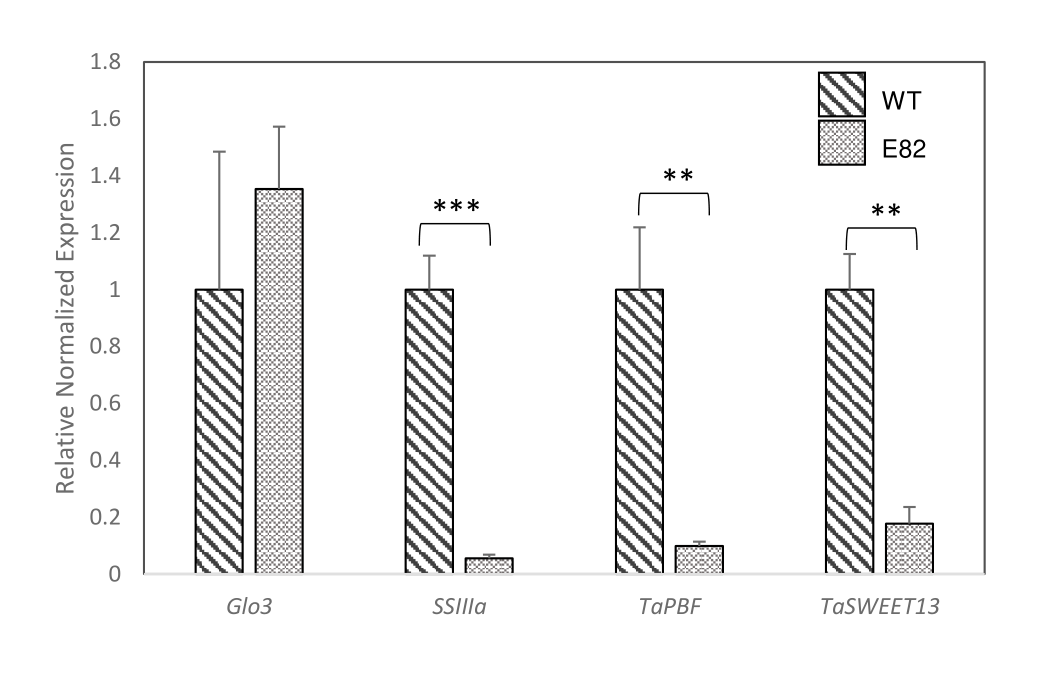


**Supplementary Figure 2.** Relative normalized expression of the wild type (WT) and E82 grains and leaves at 20 DAA for *TaPBF*, Glo3, *SSIIIa*, and *TaSWEET13* genes by qPCR. Bars indicate the standard error of three biological replicates. **, *P* < 0.01; ***, *P* < 0.001.

# Supplementary Tables

**Supplementary Table 1.** Trimming of raw reads for RNA-seq data analysis. PE reads: paired-end reads, Rep: replicate.

| **Sample** | **Number of PE reads** | **Number of clean PE reads** | **Effective rate (%)** | **Forward reads only surviving (%)** | **Reverse reads only surviving (%)** | **Dropped reads (%)** |
| --- | --- | --- | --- | --- | --- | --- |
| BW208 Leaf Rep 1 | 74,752,131 | 73,037,333 | 97.71 | 2.19 | 0.04 | 0.06 |
| BW208 Leaf Rep 2 | 71,353,011 | 69,950,624 | 98.03 | 1.85 | 0.07 | 0.05 |
| BW208 Leaf Rep 3 | 56,516,174 | 55,259,622 | 97.78 | 2.04 | 0.09 | 0.10 |
| E82 Leaf Rep 1 | 84,242,149 | 83,170,383 | 98.73 | 1.24 | 0.03 | 0.00 |
| E82 Leaf Rep 2 | 81,215,756 | 79,890,887 | 98.37 | 1.59 | 0.03 | 0.01 |
| E82 Leaf Rep 3 | 78,480,143 | 77,444,904 | 98.68 | 1.28 | 0.03 | 0.01 |
| BW208 Grain Rep 1 | 74,518,649 | 73,520,129 | 98.66 | 1.27 | 0.06 | 0.01 |
| BW208 Grain Rep 2 | 69,791,185 | 68,721,043 | 98.47 | 1.48 | 0.04 | 0.01 |
| BW208 Grain Rep 3 | 72,447,691 | 71,661,718 | 98.92 | 1.04 | 0.04 | 0.01 |
| E82 Grain Rep 1 | 66,306,206 | 66,306,206 | 99.09 | 0.86 | 0.03 | 0.01 |
| E82 Grain Rep 2 | 70,719,358 | 69,495,582 | 98.27 | 1.67 | 0.04 | 0.02 |
| E82 Grain Rep 3 | 50,926,417 | 50,231,971 | 98.64 | 1.31 | 0.04 | 0.02 |

**Supplementary Table 2.** Results of quality control of aligned reads by samtools. Parameters: default parameters. Reads are considered as the sum of forward and reverse ones. QC: quality control. Rep: replicate.

| **Sample** | **QC passed reads** | **QC failed reads** | **Number of reads secondary** | **Number of mapped reads (forward and reverse separately)** | **Rate of mapped reads (%)** | **Number of reads paired in sequencing** | **Reads properly paired (%)** | **Singletons (%)** |
| --- | --- | --- | --- | --- | --- | --- | --- | --- |
| **BW208 Leaf Rep 1** | 165,858,823 | 0 | 19,784,157 | 97,917,955 | 59.04 | 146,074,666 | 51.72 | 1.77 |
| **BW208 Leaf Rep 2** | 159,944,144 | 0 | 20,042,896 | 105,211,830 | 65.78 | 139,901,248 | 58.05 | 2.83 |
| **BW208 Leaf Rep 3** | 122,664,967 | 0 | 12,145,723 | 69,129,593 | 56.36 | 110,519,244 | 49.68 | 1.89 |
| **E82 Leaf Rep 1** | 209,745,327 | 0 | 43,404,561 | 171,003,105 | 81.53 | 166,340,766 | 74.38 | 2.33 |
| **E82 Leaf Rep 2** | 196,404,544 | 0 | 36,622,770 | 155,214,808 | 79.03 | 159,781,774 | 71.56 | 2.66 |
| **E82 Leaf Rep 3** | 195,765,253 | 0 | 40,875,445 | 160,022,010 | 81.74 | 154,889,808 | 74.76 | 2.17 |
| **BW208 Grain Rep 1** | 172,826,311 | 0 | 25,786,053 | 133,146,643 | 77.04 | 147,040,258 | 70.77 | 2.24 |
| **BW208 Grain Rep 2** | 161,795,752 | 0 | 24,353,666 | 126,835,486 | 78.39 | 137,442,086 | 72.18 | 2.38 |
| **BW208 Grain Rep 3** | 166,343,532 | 0 | 23,020,096 | 129,988,550 | 78.14 | 143,323,436 | 72.74 | 1.89 |
| **E82 Grain Rep 1** | 152,749,397 | 0 | 21,338,639 | 118,389,468 | 77.51 | 131,410,758 | 71.81 | 2.04 |
| **E82 Grain Rep 2** | 161,221,617 | 0 | 22,230,453 | 121,834,633 | 75.57 | 138,991,164 | 75.57 | 1.57 |
| **E82 Grain Rep 3** | 116,361,061 | 0 | 15,897,119 | 87,716,282 | 75.38 | 100,463,942 | 69.80 | 1.69 |

**Supplementary Table 3.** List of primers for the qPCR assay for gene expression analysis.

| Gene name | Gene ID | Primer sequence (5’-3’) | Reference |
| --- | --- | --- | --- |
| *TaPBF*^a^ | *TraesCS5A02G155900*  *TraesCS5B02G154100*  *TraesCS5D02G161000* | AGAGCAGAAGGTGGAATGCC  GTAGCGGGGCTGAGACATAC | This work. |
| *Glo3*^a^ | *TraesCS4A02G296100*  *TraesCS4A02G296000* | ATCGCCAAGATTCTCCACAC  GGACGATGGAGATGGACTTC | This work. |
| *SSIIIa*^a^ | *TraesCS1B02G119300*  *TraesCS1D02G100100*  *TraesCS1A02G091500* | TGGAAATGGAGGCTTTTCAC  TGTAGGCCTCCTTGGGTATG | This work. |
| *TaSWEET13*^b^ | *TraesCS6B02G421800*  *TraesCS6D02G367400* | CGAGTCATCAAGACCAAGAGTG  AGGCCGTAGAGGAACCAGAC | This work. |
| *CDC*^c^ | EU267938 | CAGCTGCTGACTGAGATGGA  ATGTCTGGCCTGTTGGTAGC | Giménez et al., 2011 |
| *ADP-RF*^c^ | AB050957 | TCTCATGGTTGGTCTCGATG  GGATGGTGGTGACGATCTCT | Giménez et al., 2011 |
| *RLI*^c^ | AK331207 | TTGAGCAACTCATGGACCAG  GCTTTCCAAGGCACAAACAT | Giménez et al., 2011 |

^a^ Three homeologs are amplified.

^b^ Two homeologs are amplified.

^c^ The accession number from NCBI is indicated.

**Supplementary Table 4.** Results of the alignment of each sample by kallisto. Parameters: default parameters and --pseudobam. PE: paired-end reads. Rep: replicate.

| **Sample** | **Number of clean PE reads** | **Number of aligned PE reads** | **Rate of alignment (%)** |
| --- | --- | --- | --- |
| BW208 Leaf Rep 1 | 73,037,333 | 40,356,415 | 55.25 |
| BW208 Leaf Rep 2 | 69,950,624 | 44,562,509 | 63.71 |
| BW208 Leaf Rep 3 | 55,259,622 | 29,533,590 | 53.45 |
| E82 Leaf Rep 1 | 83,170,383 | 65,734,409 | 79.04 |
| E82 Leaf Rep 2 | 79,890,887 | 61,420,333 | 76.88 |
| E82 Leaf Rep 3 | 77,444,904 | 61,251,809 | 79.09 |
| BW208 Grain Rep 1 | 73,520,129 | 55,329,460 | 75.26 |
| BW208 Grain Rep 2 | 68,721,043 | 52,879,418 | 76.95 |
| BW208 Grain Rep 3 | 71,661,718 | 54,840,952 | 76.53 |
| E82 Grain Rep 1 | 65,705,379 | 49,868,499 | 75.90 |
| E82 Grain Rep 2 | 69,495,582 | 50,894,143 | 73.23 |
| E82 Grain Rep 3 | 50,231,971 | 36,756,483 | 73.17 |

**Supplementary Table 5.** Proteomic data for grain proteins at harvest in the wild type (WT) and E82. The average of two biological replicates and the results of the t-test for the WT vs E82 comparison are represented.

|  |  | **WT** | | **E82** | | |  |
| --- | --- | --- | --- | --- | --- | --- | --- |
| **Protein** | **Enzyme** | **Number unique peptides^a^** | **SD^b^** | **Number unique peptides^a^** | **SD^b^** | **Significance of WT vs E82** | |
| ALPs | Trypsin | 93 | 7.1 | 103.5 | 6.4 | NS | |
| ATIs | Trypsin | 202 | 25.5 | 200.5 | 9.2 | NS | |
| Triticins | Trypsin | 5.5 | 2.1 | 23.5 | 0.7 | ** | |
| ω-gliadins | Chymotrypsin | 27.5 | 2.1 | 4.5 | 0.7 | ** | |
| α-gliadins | Chymotrypsin | 97.5 | 16.3 | 22 | 0 | * | |
| γ-gliadins | Chymotrypsin | 107 | 9.9 | 40.5 | 0.7 | * | |
| HMWs | Trypsin | 215.5 | 19.1 | 258.5 | 20.5 | NS | |
| LMWs | Trypsin | 150 | 11.3 | 103 | 7.1 | * | |
| Globulins | Trypsin | 29.5 | 0.7 | 57.5 | 3.5 | ** | |
| LTPs | Trypsin | 24.5 | 0.7 | 29 | 0 | * | |
| Serpins | Trypsin | 71.5 | 9.2 | 179 | 26.9 | * | |

**, P < 0.05.*

***, P < 0.01.*

****, P < 0.001.*

*NS, Non-significant.*

*^a^ Number of unique peptides: peptides with e-value < 0.05, non-redundant, included in proteins with more than 1 peptide assigned.*

*^b^ SD: standard deviation.*

**Supplementary Table 6.** Differential Gene Expression (DGE) analysis of Starch Synthesis-Related Genes (SSRGs) for the pair-wise comparison wild type (WT) vs E82.

|  |  | **Grain**  **WT vs E82** | | **Leaf**  **WT vs E82** | |
| --- | --- | --- | --- | --- | --- |
| **Gene name** | **Gene ID** | **Log_2_FC** | **FDR** | **Log_2_FC** | **FDR** |
| *SUSase* | *TraesCS7B02G063400* | -0.16 | ns | -1.57 | ***** |
| *SUSase* | *TraesCS7D02G159800* | -0.29 | ns | -1.02 | ***** |
| *APL3/AGPL1* | *TraesCS1B02G449700* | -2.36 | * | -2.27 | ***** |
| *APL3/AGPL1* | *TraesCS1D02G427400* | -2.47 | * | -1.55 | ns |
| *APS1/AGPS1/Bt2* | *TraesCS7A02G287400* | -2.17 | * | 0.01 | ns |
| *ISA3* | *TraesCS5A02G248700* | 0.26 | ns | 5.50 | ******* |
| *OsBT1-1* | *TraesCS6B02G210000* | -12.45 | *** | NA | ns |
| *OsBT1-1* | *TraesCS6D02G168200* | -11.64 | *** | NA | ns |
| *GBE1/BEIIb/Ae* | *TraesCS2A02G310300* | -5.59 | *** | 7.76 | ******* |
| *GBE1* | *TraesCS7A02G549100* | -3.08 | ** | -0.51 | ns |
| *GBE1* | *TraesCS7B02G472500* | -2.75 | ns | 9.92 | ******* |
| *SSI* | *TraesCS7B02G018600* | -1.85 | * | 0.89 | ns |
| *SSII a/SSII-3* | *TraesCS7B02G093800* | -4.80 | *** | 5.24 | ns |
| *SSII a/SSII-3* | *TraesCS7D02G190100* | -3.57 | * | NA | ns |
| *GBSSII* | *TraesCS2B02G390700* | -0.11 | ns | 2.43 | ****** |
| *GBSSI* | *TraesCS4A02G418200* | -3.53 | ** | NA | ns |
| *GBSSI/waxy* | *TraesCS7A02G070100* | -3.24 | ** | -6.54 | ******* |
| *GBSSI/waxy* | *TraesCS7D02G064300* | -3.98 | *** | NA | ns |
| *SSIIIa/SSIII-2* | *TraesCS1B02G119300* | -6.78 | ** | -2.92 | ns |
| *SPS* | *TraesCS4B02G091100* | 0.34 | ns | 1.43 | ****** |

*FC: Fold-Change.*

*FDR: False Discovery Rate.*

*ns: non-significant; *, FDR < 0.05; **, FDR < 0.01; ***, FDR < 0.001.*

*NA: Non-applicable. Genes not expressed in either the WT or the E82.*

**Supplementary Table 7.** Differential Gene Expression (DGE) analysis of well-known Transcription Factors (TFs) genes that regulate Seed Storage Proteins (SSPs) genes and Starch Synthesis-Related Genes (SSRGs) expression. Significant results (FDR < 0.05) are indicated.

|  |  | **Grain**  **WT vs E82** | |
| --- | --- | --- | --- |
| **TF name** | **Gene ID** | **Log_2_FC** | **FDR** |
| *TaFUSCA3* | *TraesCS3B02G278000* | -0.07 | ns |
| *TaFUSCA3* | *TraesCS3D02G249100* | -0.27 | ns |
| *TaFUSCA3* | *TraesCS3A02G249100* | 0.18 | ns |
| *TaPBF* | *TraesCS5A02G155900* | -6.10 | ****** |
| *TaPBF* | *TraesCS5B02G154100* | -4.98 | ns |
| *TaPBF* | *TraesCS5D02G161000* | -5.24 | ns |
| *TaSHP* | *TraesCS5A02G440400* | -0.21 | ns |
| *TaSHP* | *TraesCS5D02G447500* | 0.06 | ns |
| *TaSHP* | *TraesCS5B02G444100* | -0.36 | ns |
| *GAMYB* | *TraesCS3A02G336500* | -2.66 | ns |
| *GAMYB* | *TraesCS3B02G367500* | -0.22 | ns |
| *GAMYB* | *TraesCS3D02G329400* | -0.46 | ns |
| *MCB1* | *TraesCS1D02G220500* | -0.26 | ns |
| *MCB1* | *TraesCS1B02G232300* | -0.37 | ns |
| *MCB1* | *TraesCS1A02G218800* | -0.15 | ns |
| *MYBS3* | *TraesCS1A02G219400* | -0.34 | ns |
| *MYBS3* | *TraesCS1D02G221000* | 0.01 | ns |
| *MYBS3* | *TraesCS1B02G232800* | 0.02 | ns |
| *TBP1* | *TraesCS1A02G139600* | -0.38 | ns |
| *TBP1* | *TraesCS1B02G151700* | -0.61 | ns |
| *TBP2* | *TraesCS4D02G014000* | 0.37 | ns |
| *TBP2* | *TraesCS4B02G015700* | 0.07 | ns |
| *TBP2* | *TraesCS4A02G298000* | 0.25 | ns |
| *TaRSR1* | *TraesCS1B02G076300* | -0.50 | ns |
| *TaRSR1* | *TraesCS1A02G058400* | -0.24 | ns |
| *TaRSR1* | *TraesCS1D02G059200* | 0.10 | ns |
| *TaGBF1* | *TraesCS1A02G409800* | -0.44 | ns |
| *TaGBF1* | *TraesCS1B02G439800* | -0.65 | ns |
| *TaGBF1* | *TraesCS1D02G417100* | 0.32 | ns |
| *TabZIP229. 1* | *TraesCS7B02G114300* | 1.24 | ns |
| *TabZIP206* | *TraesCS7B02G391800* | 0.50 | ns |
| *TabZIP217.1* | *TraesCS7D02G475100* | -1.15 | ns |
| *TabZIP110* | *TraesCS4B02G175800* | -0.14 | ns |
| *TabZIP236* | *TraesCS1D02G306000* | -0.40 | ns |
| *TabZIP151* | *TraesCS5D02G447400* | 0.36 | ns |
| *TabZIP194.3* | *TraesCS7A02G488600* | 0.94 | ns |
| *TabZIP117.1* | *TraesCS4B02G178600* | 0.06 | ns |
| *TabZIP229.1* | *TraesCS7B02G114300* | 1.24 | ns |
| *TabZIP238.1* | *TraesCS7A02G207100* | 1.50 | ns |
| *TabZIP167.2* | *TraesCS5D02G178800* | -3.15 | ns |
| *TabZIP184.2* | *TraesCS6B02G193200* | -0.12 | ns |
| *TabZIP59.2* | *TraesCS3B02G404800* | 0.40 | ns |
| *TabZIP77.1* | *TraesCS3D02G365200* | -0.13 | ns |
| *TabZIP145.3* | *TraesCS5B02G142200* | -0.14 | ns |
| *TabZIP101.1* | *TraesCS4D02G230200* | 0.15 | ns |
| *TabZIP137* | *TraesCS5A02G299400* | -0.32 | ns |
| *TabZIP157.1* | *TraesCS5D02G308600* | 0.64 | ns |
| *TabZIP111* | *TraesCS4B02G113400* | -0.37 | ns |
| *TabZIP121* | *TraesCS4D02G115200* | 0.27 | ns |
| *TabZIP54.1* | *TraesCS2B02G269600* | 0.42 | ns |

*FC: Fold-Change.*

*FDR: False Discovery Rate.*

*ns: non-significant; *, FDR < 0.05; **, FDR < 0.01; ***, FDR < 0.001.*

**Supplementary Table 8.** Top 3 Gene Ontology (GO) terms enriched for each candidate transcription factor (TF) summarized from previously published GO enrichment analysis for GENIE3 network genes in Ramírez-González et al., 2018. The black boxes indicate that this is an enriched GO for this TF gene.

**Supplementary Table 9.** Complete results of Gene Ontology (GO) enrichment analysis of genes in module 13 of co-expression network WGCNA published in Ramírez-González et al. (2018). GO:MF, Molecular Function domain; GO:BP, Biological Process domain; GO:CC, Cellular Component domain.

**Supplementary Table 10.** Papain family cysteine proteases (CysProt) genes and inhibitors of CysProt genes differentially expressed (DE) in E82 leaf tissue.

|  |  | **Leaf**  **WT vs E82** | |
| --- | --- | --- | --- |
| **Gene name** | **Gene ID** | **Log_2_FC** | **FDR** |
| **CysProt inhibitor genes** |  |  |  |
| *Icy1-2* | *TraesCS1B02G322100* | -1.49 | ***** |
| *Icy1-2* | *TraesCS1D02G310300* | -1.48 | ****** |
| *Icy8* (*HvCPI8*) | *TraesCS2A02G126100* | -2.33 | ******* |
| *Icy8* (*HvCPI8*) | *TraesCS2D02G128900* | -2.23 | ******* |
| *Icy2* (*HvCPI2*) | *TraesCS5D02G502200* | 7.20 | ***** |
| **CysProt genes** |  |  |  |
| *RD19D* | *TraesCS2A02G187400* | 4.37 | ******* |
| Triticain beta (*HvPap7*) | *TraesCS2A02G545700* | -3.82 | ******* |
| *Psy1-D1* | *TraesCS3D02G232500* | 7.86 | ******* |
| *HvPap14* ortholog | *TraesCS3A02G348500* | 6.15 | ****** |
| *HvPap1* ortholog | *TraesCS5A02G326100* | -1.03 | ***** |
| NA | *TraesCS4A02G252800* | -1.26 | ***** |
| NA | *TraesCS4B02G062500* | -1.35 | ***** |
| *HvPap4* ortholog | *TraesCS5B02G138300* | 7.15 | ***** |
| *HvPap4* ortholog | *TraesCS5D02G152400* | 7.84 | ****** |

*NA: non-assigned.*

*FC: Fold-Change.*

*FDR: False Discovery Rate.*

**, FDR < 0.05; **, FDR < 0.01; ***, FDR < 0.001.*

**Supplementary Table 11.** Differentially expressed (DE) genes encoding serine-type proteases in E82 leaf against the wild type (WT).

|  | **Leaf**  **WT vs E82** | |
| --- | --- | --- |
| **Gene ID** | **Log_2_FC** | **FDR** |
| *TraesCS1A02G178200* | -1.47 | * |
| *TraesCS1A02G178300* | -1.33 | * |
| *TraesCS1A02G187300* | 6.75 | *** |
| *TraesCS1B02G110400* | -10.12 | *** |
| *TraesCS1B02G246100* | 6.91 | * |
| *TraesCS1B02G434100* | -2.97 | *** |
| *TraesCS1B02G472100* | 1.35 | * |
| *TraesCS2A02G398900* | 5.76 | * |
| *TraesCS2A02G401000* | 7.64 | ** |
| *TraesCS3A02G181400* | 5.85 | * |
| *TraesCS3A02G182700* | 6.89 | ** |
| *TraesCS3B02G211400* | 6.90 | *** |
| *TraesCS3B02G212700* | 8.18 | ** |
| *TraesCS3B02G337400* | -2.06 | ** |
| *TraesCS3B02G431900* | 8.16 | ** |
| *TraesCS4B02G352100* | -8.37 | ** |
| *TraesCS4D02G076000* | -1.28 | * |
| *TraesCS4D02G350300* | 7.33 | ** |
| *TraesCS5A02G318800* | 6.21 | * |
| *TraesCS5A02G526000* | -7.63 | * |
| *TraesCS5B02G404600* | 9.58 | *** |
| *TraesCS6A02G161600* | -1.38 | * |
| *TraesCS6A02G220500* | 1.11 | * |
| *TraesCS6B02G041200* | 7.60 | ** |
| *TraesCS6D02G319900* | -3.18 | *** |
| *TraesCS7A02G124000* | 3.82 | * |
| *TraesCS7A02G510000* | 7.42 | * |
| *TraesCS7B02G128300* | 9.54 | ** |
| *TraesCS7B02G168100* | -1.62 | ** |
| *TraesCS7D02G121200* | -1.40 | * |
| *TraesCS7D02G470100* | 8.04 | *** |
| *TraesCS1A02G178200* | -1.47 | * |
| *TraesCS1A02G178300* | -1.33 | * |
| *TraesCS1A02G187300* | 6.75 | *** |
| *TraesCS1B02G110400* | -10.12 | ******* |

*FC: Fold-Change.*

*FDR: False Discovery Rate.*

**, FDR < 0.05; **, FDR < 0.01; ***, FDR < 0.001.*

**Supplementary Table 12.** Differentially expressed (DE) genes encoding sugar and amino acid transporters in the leaf of E82.

|  |  | **Leaf**  **WT vs E82** | |
| --- | --- | --- | --- |
| **Gene name** | **Gene ID** | **Log_2_FC** | **FDR** |
| *SUT1A* | *TraesCS4A02G016400* | -1.67 | *** |
| *SUT1C* | *TraesCS4B02G287800* | -1.65 | ** |
| *SUT1D* | *TraesCS4D02G286500* | -1.60 | ** |
| *TaCWIN* | *TraesCS2B02G311900* | 5.18 | * |
| *TaCWIN* | *TraesCS2D02G293200* | 5.20 | * |
| *TaSWEET13b-6D* | *TraesCS6D02G367300* | -2.09 | *** |
| *TaSWEET13d-6B* | *TraesCS6B02G421700* | -2.57 | *** |
| *TaSWEET13e-6A* | *TraesCS6A02G382600* | -2.88 | ** |
| *TaSWEET13f-6D* | *TraesCS6D02G367400* | -2.87 | *** |
| *TaSWEET13g-6B* | *TraesCS6B02G421800* | -2.76 | ** |
| *TaSWEET13h-6B* | *TraesCS6B02G421500* | -2.94 | ** |
| *TaSWEET13i-6B* | *TraesCS6B02G421600* | -2.38 | *** |
| *TaSWEET15a-7D* | *TraesCS7D02G149000* | -10.77 | *** |
| *TaSWEET15b-7A* | *TraesCS7A02G147300* | -2.76 | ** |
| *TaSWEET15c-7B* | *TraesCS7B02G050500* | -9.18 | *** |
| *TaSWEET1a1-3B* | *TraesCS3B02G441800* | 4.76 | ** |
| *TaSWEET4a-6A* | *TraesCS6A02G218800* | -1.26 | * |
| *TaSWEET4b-6B* | *TraesCS6B02G248300* | -1.51 | ** |
| *TaSWEET6b17-7D* | *TraesCS7D02G160600* | 2.58 | * |
| *TaAAP1-1D* | *TraesCS1D02G264700* | -1.83 | * |
| *TaAAP12-4A* | *TraesCS4A02G215300* | -1.81 | ** |
| *TaAAP12-4B* | *TraesCS4B02G100800* | -1.51 | * |
| *TaAAP14-5A* | *TraesCS5A02G115100* | -1.64 | * |
| *TaAAP14-5B* | *TraesCS5B02G116100* | -1.73 | * |
| *TaAAP14-5D* | *TraesCS5D02G125700* | -2.00 | ** |
| *TaAAP15-5B* | *TraesCS5B02G120800* | -2.66 | ** |
| *TaAAP15-Un* | *TraesCSU02G134900* | -2.77 | ** |
| *TaAAP17-6D* | *TraesCS6D02G265800* | -2.47 | * |
| *TaAAP2-2A* | *TraesCS2A02G268200* | -2.73 | ** |
| *TaAAP22-7A* | *TraesCS7A02G356639* | 6.46 | ** |
| *TaAAP3-2D* | *TraesCS2D02G331800* | -2.08 | * |
| *TaAAP6-2A* | *TraesCS2A02G499800* | 9.02 | *** |
| *TaAAP8-3A* | *TraesCS3A02G388000* | 8.11 | * |
| *TaAAP9-3A* | *TraesCS3A02G388100* | -2.85 | * |
| *TaAAP9-3B* | *TraesCS3B02G420600* | -3.32 | *** |
| *TaAAP9-3D* | *TraesCS3D02G381400* | -2.76 | *** |
| *TaANT6-6D* | *TraesCS6D02G235500* | 10.55 | *** |
| *TaATLa1-3D* | *TraesCS3D02G340400* | -4.26 | ** |
| *TaATLa6-7A* | *TraesCS7A02G517100* | 6.12 | ** |
| *TaATLb2-2A* | *TraesCS2A02G052500* | 8.22 | ** |
| *TaATLb3-2D* | *TraesCS2D02G280800* | -2.42 | * |
| *TaAUX2-1B* | *TraesCS1B02G287300* | 6.51 | * |
| *TaAUX3-3A* | *TraesCS3A02G369200* | -1.51 | * |
| *TaAUX3-3B* | *TraesCS3B02G401000* | -2.00 | ** |
| *TaBAT4-3A* | *TraesCS3A02G484600* | -11.34 | *** |
| *TaCAT5-4B* | *TraesCS4B02G013800* | 9.07 | *** |
| *TaLHT3-2D* | *TraesCS2D02G402900* | 8.99 | *** |
| *TaLHT5-5A* | *TraesCS5A02G089400* | 9.68 | *** |
| *TaLHT5-5B* | *TraesCS5B02G095400* | 9.09 | *** |
| *TaProT1-2B* | *TraesCS2B02G287200* | 1.45 | * |
| *TaProT2-3A* | *TraesCS3A02G414300* | -3.29 | ** |
| *TaProT2-3B* | *TraesCS3B02G449100* | -4.17 | *** |
| *TaProT2-3D* | *TraesCS3D02G408800* | -3.76 | *** |
| *TaProT3-4A* | *TraesCS4A02G179500* | -2.01 | *** |
| *TaTTP3-4A* | *TraesCS4A02G083100* | 2.73 | *** |

*FC: Fold-Change.*

*FDR: False Discovery Rate.*

*ns: non-significant; *, FDR < 0.05; **, FDR < 0.01; ***, FDR < 0.001.*
